# Comparative Analysis of the Antioxidative and Hepatoprotective Activities of Dimethyl Diphenyl Bicarboxylate in Four Animal Models of Hepatic Injury

**DOI:** 10.3390/antiox10101508

**Published:** 2021-09-23

**Authors:** Jing-Hua Wang, Seung-Ju Hwang, Chang-Gue Son

**Affiliations:** 1 Institute of Bioscience & Integrative Medicine, Daejeon University, 75, Daedeok-daero 176, Seo-gu, Daejeon 35235, Korea; ewccwang@gmail.com (J.-H.W.); bluesea9292@naver.com (S.-J.H.); 2 Liver and Immunology Research Center, Daejeon Korean Medicine Hospital, 75, Daedeok-daero 176, Seo-gu, Daejeon 35235, Korea

**Keywords:** DDB, oxidative stress, liver injury, CCl_4_, DMN, TAA, immobilization

## Abstract

As a well-known hepatoprotective and antioxidant agent, dimethyl diphenyl bicarboxylate (DDB) has frequently been employed to remedy various liver diseases. However, it is still uncertain whether DDB exerts consistent hepatoprotective and antioxidative activities against varying degrees of hepatic damage. Therefore, DDB (100, 25, 5, or 50 mg/kg depending on the model) was administered to animals in four representative models of liver injury (CCl_4_ chemical acute model, DMN subchronic model, TAA chronic model, and restraint stress psychological acute model). Horizontal comparative analysis indicated that DDB significantly lowered the excess serum AST and ALT levels in the CCl_4_ and DMN models but not in the TAA and restraint stress models. In accordance with this result, DDB markedly reduced oxidative stress indices (hepatic MDA and ROS) but restored five main antioxidant components (GSH content, GSH-peroxidase, GSH-reductase, SOD, and catalase activity) in the CCl_4_ and DMN models. DDB failed to normalize oxidative stressors in the restraint stress-induced injury model and restore these five antioxidant components in the TAA model. Overall, our results produced a comprehensive overview of the effects of DDB on oxidative stressors and the main antioxidative components using four animal models. These findings will provide valuable clues to guide therapeutic clinical applications.

## 1. Introduction

The liver is a representative metabolic organ that is vulnerable to oxidative stress, and an imbalance in redox homeostasis has been implicated as a pivotal factor for causing numerous liver disorders [1,2,3]. Various exogenous and endogenous etiologies, such as drugs, xenobiotics and psychogenic stress, lead to liver injury via the excessive accumulation of reactive oxygen species (ROS) and overconsumption of antioxidative ability [2,4,5]. Therefore, chemical hepatotoxins, such as carbon tetrachloride (CCl_4_), dimethylnitrosamine (DMN) and thioacetamide (TAA), have been widely used to induce varying degrees of liver injury in antioxidant-related hepatoprotective animal studies [6]. Moreover, emotional stress, such as immobilization, is another a vital pathological factor that results in liver injury [5,7]. Although the underlying mechanisms are not identical, oxidative stress undoubtedly plays a crucial role in accelerating the progression of liver damage induced by all of these different pathogenic factors [1,8]. Therefore, it has long been highly regarded that antioxidants, including N-acetylcysteine, silymarin, and others, are a promising approach to relieve various acute and chronic liver injuries [9,10,11].

Many natural plants and their active compounds possess potent antioxidant effects to improve liver injury [12], such as milk thistle (silymarin) and turmeric (curcumin) [13,14]. In Eastern Asia, *Schisandra chinensis*, also named five flavor berry, is a famous edible and medicinal herb that has been traditionally used for remedying various disorders, including amelioration of liver function [15]. Pharmacological studies have revealed that schizandrin, a main active ingredient in *S. chinensis*, exerts anti-inflammatory and antioxidative effects [16,17]. Dimethyl diphenyl bicarboxylate (DDB) is a pharmaceutical intermediate derivative that is used to artificially synthesize active compounds, such as schizandrins C and D [18]. Commonly, natural herbs are difficult to ensure the stable effect due to the diversity of ingredients and non-standardization, and in terms of time and cost, separation of the active compound from herb is also not easy. Thus, DDB is a promising approach as compared to *Schisandra chinensis* and its active compounds for clinical application. In addition, DDB effectively ameliorated serum aminotransferase, oxidative stress-associated parameters, and liver fibrosis in previous studies [18,19,20]. Owing to its quick effects, low price and easy synthesis by using gallic acid, DDB has widely been applied as a hepatoprotective and antiviral therapeutic agent to cure various liver disorders in Asian countries, such as south Korea, China, and Indonesia [21,22,23,24]. However, a pilot clinical study showed that DDB rapidly decreased the level of serum alanine aminotransferase (ALT) to the normal range but not aspartate aminotransferase (AST) or gamma-glutamyl transferase (GGT), and DDB also did not improve hepatic histopathological alterations in patients with chronic hepatitis [23].

Although most evidence suggests that DDB is effective against liver injury, it is still unclear whether DDB exerts consistent hepatoprotective and antioxidative effects with respect to the various degrees and types of hepatic injury. Therefore, in the present study, we evaluated the hepatoprotective capacity of DDB and compared its antioxidative characteristics using four typical hepatic injury models that have different degrees of injury and different etiologies.

## 2. Materials and Methods

### 2.1. Animal Experiments

**Figure 1:**
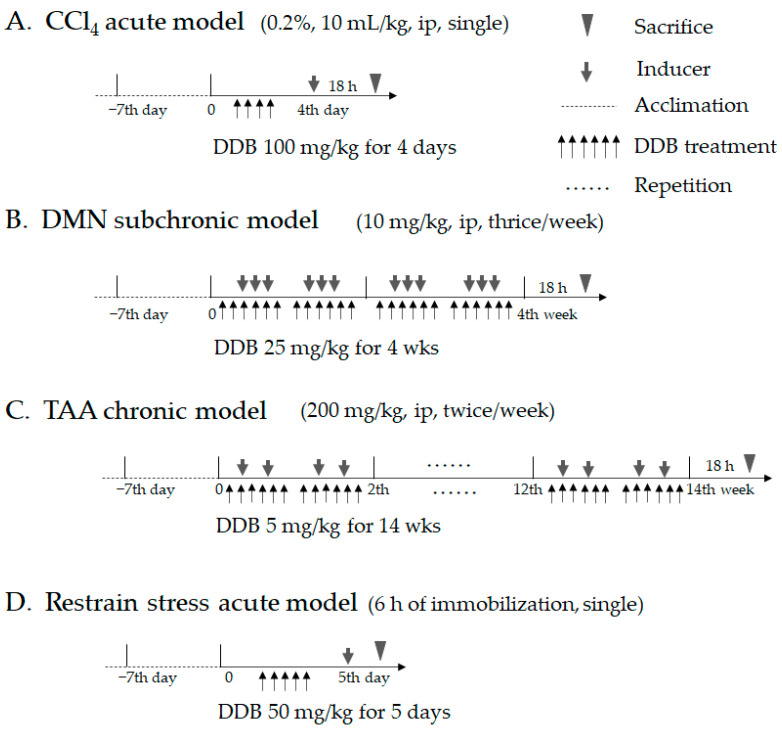
Experimental schedule of the four liver injury models. The experimental schedule scheme regarding the CCl_4_ acute model (**A**), DMN subchronic model (**B**), TAA chronic model (**C**), and restraint stress model (**D**) are illustrated in detail.

For the acute liver injury models, forty-eight male BALB/c mice (8 weeks old) were purchased from Daehan-Biolink (Chung-buk, Korea). For the subchronic and chronic liver injury model, forty-eight Sprague Dawley (SD) male rats (6 weeks old) were obtained from Orient Bio (Gyeonggi-do, Korea). After one week of acclimation, the animals were divided into 3 groups, with 8 animals in each group for each experiment. For the chemically induced acute model, BALB/c mice were treated with 0.2% carbon tetrachloride (Junsei, Tokyo, Japan) via a single ip injection. For the subchronic liver injury model, SD rats were injected intraperitoneally with 10 mg/kg DMN for 3 weeks. For the chronic liver injury model, SD rats were treated with 200 mg/kg TAA for 14 weeks. For the psychological acute liver injury model, all four limbs of BALB/c mice were tightly fixed in to a grid with quartz-pasted tape for 6 h. DDB was orally administered at doses of 100 mg/kg, 25 mg/kg, 5 mg/kg and 50 mg/kg in the chemical acute, subchronic, chronic and psychological acute liver injury models, respectively. Detailed information on the four experimental designs and schedules is shown in Table 1 and Figure 1.

All of the animal experiments were performed strictly on the basis of the Guide for the Care and Use of Laboratory Animals (National Research Council, Institute of Laboratory Animal Research, Commission on Life Sciences, USA; National Academy Press: Washington, DC, USA, 1996). The experimental design and protocols were approved by the Institutional Animal Care and Use Committee of Daejeon University with approval number DJUARB-2010012.

### 2.2. Serum Aminotransferase Determination

Abdominal aorta blood was collected by syringe under ketamine anesthesia. After 1 *h* of clotting at room temperature (RT), serum was separated by centrifugation at 3000× *g* for 15 min and stored at −70 °C for aminotransferase analysis. The serum levels of aspartate transaminase (AST) and alanine transaminase (ALT) were determined automatically using an AU480 chemistry analyzer (Beckman Coulter, Brea, CA, USA) and commercial reagents (OSR6109 for AST and OSR6107 for ALT; Beckman Coulter, Brea, CA, USA).

### 2.3. Hepatic Tissue Preparation for Hepatic Oxidative Stress-Related Parameter Determination

Fresh liver tissue was homogenized with RIPA (radioimmunoprecipitation assay) lysis buffer with a FastPrep-24 homogenizer (MP Biomedicals, Solon, OH, USA) to estimate hepatic oxidative stress and antioxidant-associated parameters. The homogenate was centrifuged at 14,000× *g* for 15 min at 4 °C, and then the supernatant was transferred into a new Eppendorf tube and stored at −70 °C for further determination.

### 2.4. Hepatic Total Reactive Oxygen Species Determination

Excessive production of reactive oxygen species (ROS) is a primary marker of oxidative stress [25]. To evaluate the status of liver oxidative stress, the ROS levels in liver tissues were determined by Hayashi’s method [26]. In brief, each liver tissue sample (5 μL) was mixed with 0.1 M sodium acetate buffer (pH 4.8, 140 μL). After 5 min of incubation at 37 °C, 100 μL of a mixture of 10 mM N, N-diethyl-para-phenylenediamine (DEPPD) and 4.37 μM ferrous sulfate (1:25, *v*/*v*) was added, and after further incubation at 37 °C for 1 min, the hepatic ROS level was determined by measuring the absorbance at 505 nm. Hydrogen peroxide (H_2_O_2_) was used to generate a standard curve for calculations.

### 2.5. Hepatic Lipid Peroxidation Determination

The level of liver lipid peroxidation was evaluated by thiobarbituric acid reactive substances (TBARS) according to our previous method [27]. Briefly, liver tissue samples from identical regions were homogenized with 1.15% potassium chloride. Liver homogenate (100 μL) was mixed with 0.02% trichloroacetic acid (TCA; 500 μL), sulfuric acid (500 μL) and 20 mg/dl thiobarbituric acid (TBA; 600 μL), heated at 100 °C for 60 min, cooled at 4 °C for 15 min, centrifuged to remove the supernatant, and 300 μL of n-butanol was added for vigorous vortexing. After centrifugation at 3000× *g* for 10 min, the absorbance of the supernatant was detected at 531 nm using a spectrophotometer (Molecular Devices, Sunnyvale, CA, USA). The hepatic malondialdehyde (MDA) content was calculated and compared with the standard curve using 1,1,3,3-tetraethoxypropane (TEP).

### 2.6. Hepatic Glutathione and Glutathione Peroxidase and Glutathione Reductase Determination

The hepatic glutathione (GSH) content was evaluated according to previous method [28]. Briefly, 50 μL of each liver tissue sample was mixed with 80 μL of a mixture solution of DTNB/NADPH (1:7 (*v*/*v*); 4 mM DTNB and 0.3 mM NADPH). After 0.06 U of GSH reductase solution was added, the absorbance was measured at 405 nm. Glutathione peroxidase (GSH-px) and glutathione reductase (GSH-red) were determined using the glutathione peroxidase cellular activity assay kit and glutathione reductase assay kit, respectively (Sigma, St. Louis, MO, USA). The above assays were performed strictly following the manufacturer’s instructions.

### 2.7. Hepatic Superoxide Dismutase Determination

Hepatic superoxide dismutase (SOD) activity was measured using a commercial SOD assay kit (Dojindo Laboratories, Kumamoto, Japan). One unit of SOD activity was defined as the quantity of enzyme required to inhibit the reduction reaction of the highly water-soluble tetrazolium salt, WST-1 (2-(4-iodophenyl)-3-(4-nitrophenyl)-5-(2,4-disulfophenyl)-2H-tetrazolium, monosodium salt) with superoxide anions. According to the manufacturer’s technical manual, the absorbance was measured at 450 nm. Lyophilized bovine erythrocyte SOD was applied to generate a standard curve for comparison.

### 2.8. Hepatic Catalase Determination

Hepatic catalase activity was determined using Wheeler’s method [29]. Briefly, phosphatase buffer (30 μL, 250 mM, pH 7.0), methanol (30 μL, 12 mM) and H_2_O_2_ (30 μL, 44 mM) were mixed with prepared liver tissue samples in 96-well plates. After 10 min of incubation at 22 °C, the reaction was terminated by adding Purpald solution (90 μL, 22.8 mM Purpald in 2 N potassium hydroxide). After mixing the potassium peroxide solution (30 μL, 65.2 mM in 0.5 N potassium hydrate), the mixture was incubated for 10 min at 22 °C. The absorbance of the purple formaldehyde adduct was measured at 550 nm.

### 2.9. Hematoxylin and Eosin Staining

Formalin-fixed hepatic tissues were embedded in paraffin wax using a HistoCore Arcadia H embedding station (Leica Biosystems, Wetzlar, Germany). Then, 5 μm thick sections were cut with a microtome (Leica RM2235, Nussloch, Germany), and silane-coated micro slides (5116-20F, MUTO PURE Chemicals, Tokyo, Japan) were used to affix the tissue sections. The prepared hepatic tissue sections were stained with hematoxylin (Merck, Boston, MA, USA), counterstained with eosin (Sigma-Aldrich, St. Louis, MO, USA) and mounted in Canada balsam. Finally, the tissue sections were observed with an Olympus IX71 microscope (Olympus, Tokyo, Japan) and photographed using an Olympus DP74 digital camera (Olympus, Tokyo, Japan).

### 2.10. Statistical Analysis

The results are expressed as the mean ± standard deviation and were normalized to the normal group. The statistical package for science software (SPSS, 19.0 version, Chicago, IL, USA) was applied for statistical analyses. The statistical significance of differences was analyzed using one-way analysis of variance (ANOVA) followed by an unpaired Student’s *t*-test. A *p* value less than 0.05 was considered to indicate a significant difference.

## 3. Results

### 3.1. Comparison of the Serum Aminotransferases and AST to ALT Ratio in Four Liver Injury Models

**Figure 2:**
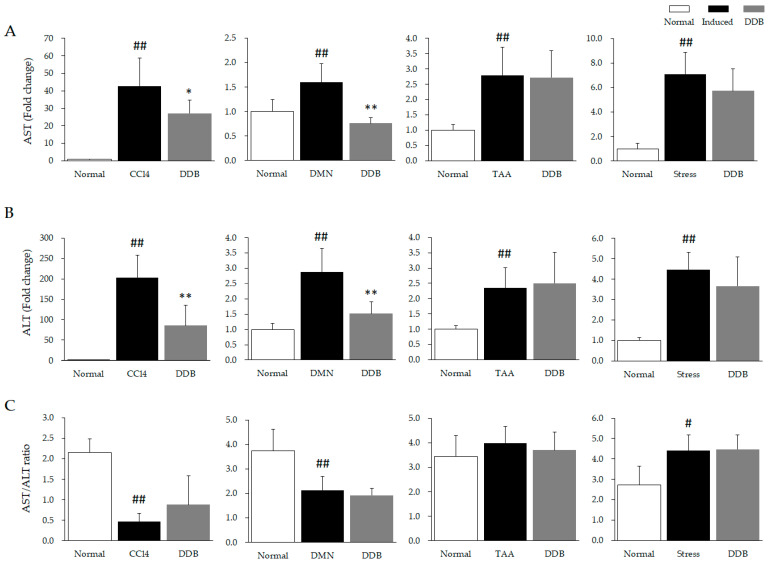
Comparison of serum aminotransferases and the AST/ALT ratio in four liver injury models. Serum AST (**A**) and ALT (**B**) levels from four types of liver injury models were examined by an AU480 chemistry analyzer, and the AST/ALT ratios (**C**) were calculated and horizontally compared. ^#^ *p* < 0.05 and ^##^ *p* < 0.01 compared with the normal group. * *p* < 0.05 and ** *p* < 0.01 compared with the corresponding control.

Both CCl_4_, DMN, and TAA treatment and restraint stress significantly elevated the serum AST (*p* < 0.01) and ALT (*p* < 0.01) levels as compared to normal (Figure 2A,B). However, DDB administration markedly attenuated these excessive AST (CCl_4_: *p* < 0.05, DMN, *p* < 0.01, Figure 2A) and ALT (CCl_4_: *p* < 0.01, DMN, *p* < 0.01, Figure 2B) in the CCl_4_ and DMN models but not in the TAA and restraint stress models.

Injection of CCl_4_ and DMN distinctly lowered the AST to ALT ratio (AAR) as compared to normal (CCl_4_: *p* < 0.01, DMN, *p* < 0.01), yet acute restraint stress noticeably increased the AAR (*p* < 0.05), and no obvious alteration was observed in the TAA model (Figure 2C). In addition, the significant alteration of AARs was not found in all DDB groups compared to each corresponding control.

### 3.2. Comparison of Hepatic ROS Activity and Lipid Peroxidation in Four Liver Injury Models

**Figure 3:**
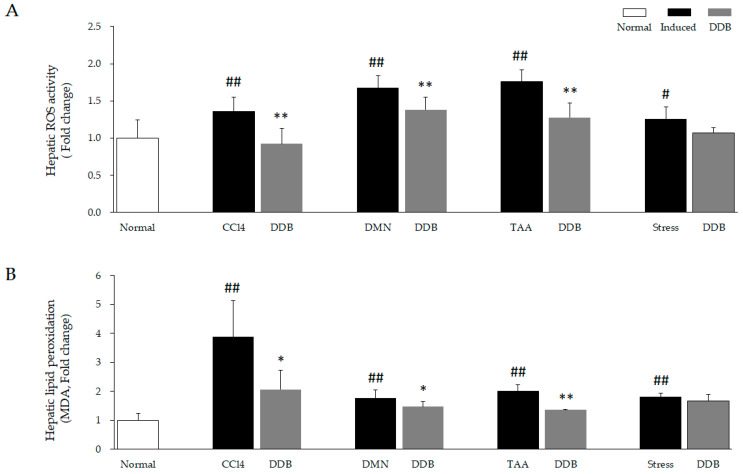
Comparison of hepatic ROS activity and lipid peroxidation in four liver injury models. Two oxidative stress parameters, reactive oxygen species (**A**) and malondialdehyde (MDA, a marker of lipid peroxidation) (**B**), were determined in prepared liver tissue samples. ^#^ *p* < 0.05 and ^##^ *p* < 0.01 compared with the Normal group. * *p* < 0.05 and ** *p* < 0.01 compared with the corresponding control.

Four liver injury inducers, including CCl_4_, DMN, TAA, restraint stress, markedly increased productions of ROS and MDA in liver tissue (CCl_4_: *p* < 0.01, DMN, *p* < 0.01, TAA: *p* < 0.01, Stress, *p* < 0.05 or *p* < 0.01, Figure 3B) as compared to normal, whereas DDB significantly reduced these alterations in three hepatoxin models (CCl_4_: *p* < 0.05 or *p* < 0.01, DMN, *p* < 0.05 or *p* < 0.01, TAA: *p* < 0.01), but not in the psychological stress model.

### 3.3. Comparison of Hepatic GSH System in Four Hepatic Injury Models

**Figure 4:**
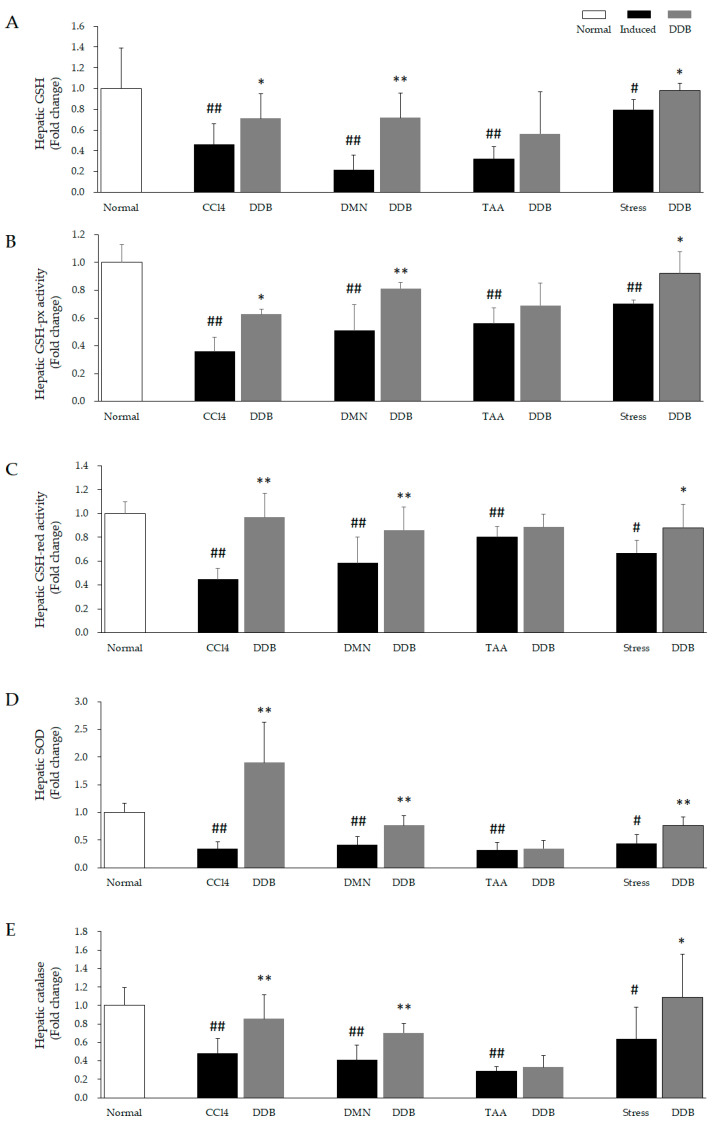
Comparison of the main hepatic antioxidative parameters in four liver injury models. Five main antioxidative parameters, GSH (**A**), GSH-px (**B**), GSH-red (**C**), SOD (**D**), and catalase (**E**), were determined in prepared liver tissue samples, as described in the Materials and Methods section. ^#^ *p* < 0.05 and ^##^ *p* < 0.01 compared with the normal group. * *p* < 0.05 and ** *p* < 0.01 compared with the corresponding control.

The content of total hepatic GSH and activities of hepatic GSH-px and GSH-red were significantly lowered in the CCl_4_ (*p* < 0.01), DMN (*p* < 0.01), TAA (*p* < 0.01), restraint stress (*p* < 0.05) group as compared to the normal group. Interestingly, three chemical hepatoxic inducers (CCl_4_, DMN and TAA) triggered greater reduction of the total GSH content in liver than psychological stress. Moreover, DDB distinctly restored the attenuation of GSH system in CCl_4_ (*p* < 0.05 or *p* < 0.01), DMN (*p* < 0.01), restraint stress (*p* < 0.05) model, but not in the TAA model (Figure 4A–C).

### 3.4. Comparison of Hepatic SOD and Catalase in Four Hepatic Injury Models

The activities of SOD and catalase in liver tissue was noticeably reduced by CCl_4_ (*p* < 0.01), DMN (*p* < 0.01), TAA (*p* < 0.01), restraint stress (*p* <0.01) treatment as compared to normal. While similar to GSH system, DDB also significantly restored the reduction of these two antioxidative enzymes activity in in CCl_4_ (*p* < 0.01), DMN (*p* < 0.01), restraint stress (*p* < 0.05 or *p* < 0.01) model, but not in TAA model (Figure 4D,E).

### 3.5. Comparison of Liver Histopathological Change in Four Hepatic Injury Models

**Figure 5:**
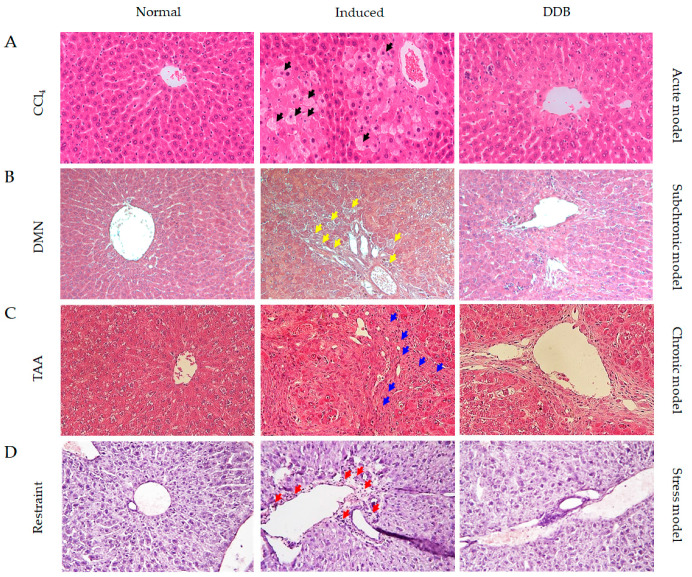
Comparison of hepatic lipid peroxidation and ROS activity in four liver injury models. Formalin-fixed liver tissues from the CCl_4_ acute model (**A**), black arrows indicated hepatocyte swelling, DMN subchronic model (**B**), yellow arrows indicated bile duct hyperplasia, TAA chronic model (**C**), blue arrows indicated collagen deposition and restraint stress model (**D**), red arrows showed neutrophil infiltration were embedded in paraffin, sectioned with a microtome, stained with hematoxylin and eosin and mounted in Canada balsam. Histopathological analysis was performed with an Olympus IX71 microscope and an Olympus DP74 camera.

Single CCl_4_ injection obviously induced hepatocyte swelling and coagulation necrosis (Figure 5A). Injection of DMN triggered slight collagen deposition and bile duct hyperplasia (Figure 5B). However, scar tissue formation led to the hepatic architecture distortions in the TAA chronic liver injury model (Figure 5C). Intriguingly, noticeable neutrophil infiltration was found in the restraint stress model (Figure 5D), whereas DDB perceptibly improved these histopathological alterations with varying degrees in four hepatic injury models.

## 4. Discussion

In mammals, the liver is a critical organ that is easily injured to different degrees by exposure to various etiologies [30,31]. Serum aminotransferases are commonly used as markers for assessing the severity of liver injury in the clinic [32]. When acute liver injury occurs, a large amount of AST and ALT are released into plasma from the damaged hepatocyte cytoplasm (or mitochondria for AST) [33]. However, AST and ALT show low sensitivity when evaluating chronic liver injury because of a significant decline in hepatocyte numbers and function due to excessive long-term damage [34]. In the present study, a single CCl_4_ injection induced a substantial increase in serum AST and ALT levels, increasing 42- and 200-fold, respectively, compared to normal levels (Figure 2A,B). However, DMN and TAA injection (subchronic and chronic model, respectively) mildly elevated the levels of AST (DMN, 1.6-fold; TAA, 2.8-fold) and ALT (DMN, 2.9-fold; TAA, 2.4-fold) compared to the acute CCl_4_ model (Figure 2A,B). In addition, psychogenic acute liver injury triggered by restraint stress caused relatively large elevations in AST (7.1-fold) and AST (4.5-fold) levels compared to the DMN and TAA models. DDB administration markedly attenuated these excessive AST and ALT in the CCl_4_ and DMN models, but not in the TAA and stress models (Figure 2A,B). This might suggest that DDB is more appropriate for ameliorating hepatotoxin-induced short-term liver injury than long-term or psychogenic injury. Moreover, different medication time was employed in the four liver injury models (pretreatment in CCl_4_ and restrain stress acute model, simultaneous treatment in DMN sub-chronic and TAA chronic model) due to the rapidly spontaneous recovery, a limitation of acute animal liver injury model. Therefore, pretreatment or simultaneous treatment of DDB is not a decisive factor for exerting hepatoprotective effect. We cannot conclude that the DDB’s prophylactic effect is superior to therapeutic effect in all type of the liver injury model.

The AST to ALT ratio (AAR), also known as the De-Ritis ratio, has frequently been utilized as a biomarker for assisting in the diagnosis or prognosis of various hepatic diseases in the clinic. For example, in the case of humans, AAR < 1 implies chronic injury, AAR > 1 indicates acute injury, and AAR > 2 suggests alcoholic liver disease [35,36]. However, much evidence has revealed that the normal AAR value is greater than two in murine because of the shorter half-life of ALT and higher activity of AST compared to humans [37,38,39]. Our data indicated that CCl_4_ and DMN significantly lowered the AAR (CCl_4_: *p* < 0.01, DMN, *p* < 0.01) compared to normal; in contrast, acute restraint stress noticeably increased the AAR (stress, *p* < 0.05), and no obvious alterations were observed in the TAA model (Figure 2C). A possible explanation for these results is that ALT is more specific and sensitive than AST in liver tissue [35], and CCl_4_ and DMN are dominantly metabolized by cytochrome P450 in the liver [40,41]; thus, ALT was increased more intensely than AST in the acute (CCl_4_) and subchronic (DMN) models. However, restraint stress more significantly enhances AST than ALT, which is understood by the fact that psychogenic stress can widely influence multiple organs of the body [42], unlike xenobiotic hepatotoxins, such as CCl_4_ and DMN. Interestingly, DDB did not notably alter the AAR in any of the four models compared to the corresponding control group. This result suggests that DDB simultaneously affects the release and/or clearance of AST and ALT in the CCl_4_ and DMN models and slightly influence liver injury itself in the chronic and psychogenic liver injury model.

On the other hand, the above results were overall in accordance with the oxidative stress parameter measurements (Figure 3A,B) rather than the antioxidant components (Figure 4A–E). ROS are the strongest oxidative stressors, and they play a pivotal role in the pathogenesis of liver injury [1]. Various environmental factors, such as chemicals and psychological stress, can induce substantial ROS accumulation in liver [43,44]. Sequentially, excessive ROS causes lipid peroxidation, which is the oxidative degradation of lipids in the cell membrane [45]. Hence, inhibition of the lipid peroxidative process is regarded as an effective approach for preventing and treating liver injury [46]. As we expected, dramatic increases in hepatic ROS and lipid peroxidation (as presented by MDA measurements) were observed in each of the four injury models, whereas DDB markedly reduced these alterations in three models, with the exception of the psychological stress model (Figure 3A,B). Overall, the effects of DDB on oxidative stressors were more predominant in the CCl_4_-induced acute injury model than in the other three models. These results of DDB with respect to ROS and lipid peroxidation are vital explanations for the hepatoprotective effects of DBB that have been found in previous animal and clinical studies [47,48,49].

Furthermore, GSH is a ubiquitous tripeptide that primarily plays an antioxidant role by preventing oxidative stress through the neutralization of free radicals [50]. In clinic, N-acetylcysteine, a precursor of GSH, has already been approved by the US Food and Drug Administration (FDA) to remedy acetaminophen-induced hepatotoxicity [51]. A previous animal study revealed that DDB exerts hepatoprotective activity potentially by increasing hepatic GSH regeneration [18]. Moreover, GSH- and GSH-related enzymes, GSH-px and GSH-red, cooperatively accelerated hydrogen peroxide elimination to prevent excessive ROS generation and maintain cellular redox homeostasis [52]. In addition, SOD and catalase are antioxidant enzymes that can synergistically convert free radicals into water and molecular oxygen [53]. Treatment with a synthetic SOD-catalase mimetic effectively attenuates tissue injury due to oxidative stress [54,55]. In our study, DDB noticeably recovered the excessive consumption of hepatic GSH, GSH-px, GSH-red, SOD and catalase in the CCl_4_, DMN and stress models (Figure 4A–E). However, unlike oxidative stressors, these five main antioxidant components were not prominently restored in the TAA chronic model (Figure 4A–E). A conceivable reason for this result is that these antioxidant components (especially SOD and catalase) are mostly exhausted in the liver due to the continuous generation of TAA metabolic intermediates (such as TAA-S-oxide) for up to 14 weeks. In addition, as a comparison with human recommended doses from the Korean FDA (75 to 450 mg/day, conversion of human dose to rat equivalent dose is 7.5 to 45 mg/kg/day [56]), the relatively low dosage of DDB administered here (5 mg/kg/day) might be the cause for the lack of significant changes in the five antioxidative components in the TTA model. This low dosage of DDB could also be linked to the lack of improvement in AST and ALT levels in the chronic TAA model. In general, the principal concept of oxidative injury is the imbalance between oxidative stressors and antioxidative components [57]. Accordingly, we can summarize the hepatoprotective effects of DDB in the CCl_4_ and DMN models as restoring the alterations to the levels of oxidative stressors and antioxidative components, but the lack of effects observed in the other two models suggested a failure in reestablishing this balance.

Along with the consistent results above, we confirmed the histological improvements promoted by DDB in CCl_4_ induced noticeable hepatocyte swelling (black arrows) and coagulation necrosis (Figure 5A), and DMN triggered mild collagen deposition and bile duct hyperplasia (yellow arrows) (Figure 5B). However, DDB treatment did not notably attenuate the severe distortions in the hepatic architecture characterized by excessive accumulation of extracellular matrix (blue arrows) in the TAA-induced chronic injury model (Figure 5C). Finally, obvious neutrophil infiltration (red arrows) was observed in the liver parenchyma under restraint stress conditions (Figure 5D). Notably, many clinical studies have revealed that DDB significantly normalizes the release of ALT but has no significant effect on the histologic or fibrotic changes in patients with chronic viruses [23]. We herein compared the hepatoprotective efficacies of DDB among four liver injury models; however, our study has some limitations. Firstly, the present study employed three xenobiotic-induced injury models and one psychogenic stress-induced injury model, which are thought to have difficulty reflecting clinically frequent disorders, such as viral-, alcohol- and nutrition overload-related liver injuries. Another limitation is that different dosages of DDB were used for treatment in the four injury models, even though we designed our study according to previously reported articles, which suggest high doses for acute injury and low doses for chronic injury [20,57].

## 5. Conclusions

Taken together, we suppose that DDB has a pharmacological activity to protect the liver from oxidative injury, especially against short-term hepatoxin-related liver damage, as it showed better results than those observed under chronic and psychological conditions. Even though there are some limitations to our study, our results produced a comprehensive overview of DDB treatment on oxidative stressors and the main antioxidant components in four representative animal models, which will provide a useful reference for guiding rational clinical medications.

## Figures and Tables

**Table 1:** Experimental design of four liver injury animal models.

Model Type	Acute Liver Injury			Subchronic Liver Injury			Chronic Liver Injury			Psychological Acute Liver Injury		
Groups	Nor	CCl_4_	DDB	Nor	DMN	DDB	Nor	TAA	DDB	Nor	Stress	DDB
Animal	BALB/c mice (8 in each group)			SD rats (8 in each group)			SD rats (8 in each group)			BALB/c mice (8 in each group)		
Inducer (Frequency)	−	CCl_4_ (0.2% in olive oil, ip, single)		−	DMN (10 mg/kg, ip, thrice/week, 3 weeks)		−	TAA (200 mg/kg, ip, twice/week, 14 weeks)		−	Restraint (6 h of immobilization, single)	
DDB (Dosage)	−	−	100 mg/kg (q.d., 4 days)	−	−	25 mg/kg (q.d., 3 weeks)	−	−	5 mg/kg (q.d., 14 weeks)	−	−	50 mg/kg (q.d., 5 days)

Nor: normal, CCl_4_: carbon tetrachloride, DDB: dimethyl diphenyl bicarboxylate, ip: intraperitoneal injection, q.d.: quaque die, DMN: dimethylnitrosamine, SD: Sprague Dawley, TAA: thioacetamide.

## Data Availability

Data is available within the article.

## References

[1] Cichoż-Lach H., Michalak A. (2014). Oxidative stress as a crucial factor in liver diseases. World J. Gastroenterol..

[2] Li S., Tan H.Y., Wang N., Zhang Z.J., Lao L., Wong C.W., Feng Y. (2015). The Role of Oxidative Stress and Antioxidants in Liver Diseases. Int. J. Mol. Sci..

[3] Zheng J., Yuan Q., Zhou C., Huang W., Yu X. (2021). Mitochondrial stress response in drug-induced liver injury. Mol. Biol. Rep..

[4] Sanchez O., Arnau A., Pareja M., Poch E., Ramirez I., Soley M. (2002). Acute stress-induced tissue injury in mice: Differences between emotional and social stress. Cell Stress Chaperones.

[5] McGill M.R., Jaeschke H. (2019). Animal models of drug-induced liver injury. Biochim. Biophys. Acta. Mol. Basis Dis..

[6] Sahin E., Gumuslu S. (2007). Immobilization stress in rat tissues: Alterations in protein oxidation, lipid peroxidation and antioxidant defense system. Comp. Biochem. Physiol. C Toxicol. Pharmacol..

[7] Kim S.H., Oh D.S., Oh J.Y., Son T.G., Yuk D.Y., Jung Y.S. (2016). Silymarin Prevents Restraint Stress-Induced Acute Liver Injury by Ameliorating Oxidative Stress and Reducing Inflammatory Response. Molecules.

[8] Shrestha D.B., Budhathoki P., Sedhai Y.R., Adhikari A., Poudel A., Aryal B., Baniya R. (2021). N-acetyl cysteine versus standard of care for non-acetaminophen induced acute liver injury: A systematic review and meta-analysis. Ann. Hepatol.

[9] Casas-Grajales S., Muriel P. (2015). Antioxidants in liver health. World J. Gastrointest. Pharmacol. Ther..

[10] Arauz J., Ramos-Tovar E., Muriel P. (2016). Redox state and methods to evaluate oxidative stress in liver damage: From bench to bedside. Ann. Hepatol..

[11] Kwak K.G., Wang J.H., Shin J.W., Lee D.S., Son C.G. (2011). A traditional formula, Chunggan extract, attenuates thioacetamide-induced hepatofibrosis via GSH system in rats. Hum. Exp. Toxicol..

[12] Surai P.F. (2015). Silymarin as a Natural Antioxidant: An Overview of the Current Evidence and Perspectives. Antioxidants.

[13] Farzaei M.H., Zobeiri M., Parvizi F., El-Senduny F.F., Marmouzi I., Coy-Barrera E., Naseri R., Nabavi S.M., Rahimi R., Abdollahi M. (2018). Curcumin in Liver Diseases: A Systematic Review of the Cellular Mechanisms of Oxidative Stress and Clinical Perspective. Nutrients.

[14] Kopustinskiene D.M., Bernatoniene J. (2021). Antioxidant Effects of *Schisandra chinensis* Fruits and Their Active Constituents. Antioxidants.

[15] Cui L., Zhu W., Yang Z., Song X., Xu C., Cui Z., Xiang L. (2020). Evidence of anti-inflammatory activity of Schizandrin A in animal models of acute inflammation. Naunyn-Schmiedebergs Arch. Pharmacol..

[16] Hu D., Cao Y., He R., Han N., Liu Z., Miao L., Yin J. (2012). Schizandrin, an antioxidant lignan from *Schisandra chinensis*, ameliorates Abeta1-42-induced memory impairment in mice. Oxid. Med. Cell. Longev..

[17] Ip S.P., Yiu H.Y., Ko K.M. (2000). Differential effect of schisandrin B and dimethyl diphenyl bicarboxylate (DDB) on hepatic mitochondrial glutathione redox status in carbon tetrachloride intoxicated mice. Mol. Cell. Biochem..

[18] Gao M., Zhang J., Liu G. (2005). Effect of diphenyl dimethyl bicarboxylate on concanavalin A-induced liver injury in mice. Liver Int..

[19] Kang K.W., Kim Y.G., Kim C.W., Kim S.G. (2002). The anti-fibrogenic effect of a pharmaceutical composition of [5-(2-pyrazinyl)-4-methyl-1,2-dithiol-3-thione] (oltipraz) and dimethyl-4,4′-dimethoxy-5,6,5′,6′-dimethylene dioxybiphenyl-2,2′-dicarboxylate (DDB). Arch. Pharmacal Res..

[20] Azzam H.S., Goertz C., Fritts M., Jonas W.B. (2007). Natural products and chronic hepatitis C virus. Liver Int..

[21] Kim H.S., Lee S.T., Kim D.G., Ahn D.S. (1996). A study on the efficacy and safety of dipheny-dimethyl-dicarboxylate in patients with chronic liver disease. Korean J. Hepatol..

[22] Huber R., Hockenjos B., Blum H.E. (2004). DDB treatment of patients with chronic hepatitis. Hepatology.

[23] Akbar N., Tahir R.A., Santoso W.D., Soemarno, Sumaryono, Noer H.M., Liu G. (1998). Effectiveness of the analogue of natural Schisandrin C (HpPro) in treatment of liver diseases: An experience in Indonesian patients. Chin. Med. J..

[24] Shankar K., Mehendale H.M., Wexler P. (2014). Oxidative Stress. Encyclopedia of Toxicology.

[25] Hayashi I., Morishita Y., Imai K., Nakamura M., Nakachi K., Hayashi T. (2007). High-throughput spectrophotometric assay of reactive oxygen species in serum. Mutat. Res..

[26] Wang J.H., Shin J.W., Son J.Y., Cho J.H., Son C.G. (2010). Antifibrotic effects of CGX, a traditional herbal formula, and its mechanisms in rats. J. Ethnopharmacol..

[27] Han J.-M., Kim H.-G., Choi M.-K., Lee J.-S., Park H.-J., Wang J.-H., Lee J.-S., Son S.-W., Hwang S.-Y., Son C.-G. (2012). Aqueous extract of Artemisia iwayomogi Kitamura attenuates cholestatic liver fibrosis in a rat model of bile duct ligation. Food Chem. Toxicol..

[28] Wheeler C.R., Salzman J.A., Elsayed N.M., Omaye S.T., Korte D.W. (1990). Automated assays for superoxide dismutase, catalase, glutathione peroxidase, and glutathione reductase activity. Anal. Biochem..

[29] Liu Y., Meyer C., Xu C., Weng H., Hellerbrand C., ten Dijke P., Dooley S. (2013). Animal models of chronic liver diseases. Am. J Physiol. Gastrointest. Liver Physiol..

[30] Vento S., Cainelli F. (2020). Acute liver failure. Lancet.

[31] Gopal D.V., Rosen H.R. (2000). Abnormal findings on liver function tests. Interpreting results to narrow the diagnosis and establish a prognosis. Postgrad. Med..

[32] Giannini E.G., Testa R., Savarino V. (2005). Liver enzyme alteration: A guide for clinicians. CMAJ.

[33] Johnston D.E. (1999). Special considerations in interpreting liver function tests. Am. Fam. Physician..

[34] Rej R. (1989). Aminotransferases in disease. Clin. Lab. Med..

[35] Nanji A.A., French S.W., Mendenhall C.L. (1989). Serum aspartate aminotransferase to alanine aminotransferase ratio in human and experimental alcoholic liver disease: Relationship to histologic changes. Enzyme.

[36] Lee R.C. (1989). Laboratory Animal Medicine.

[37] Chen X., Tan F., Yi R., Mu J., Zhao X., Yang Z. (2018). Effects of Lactobacillus on Mice with Diabetes Induced by High-Fat Diet with Streptozotocin (STZ). Appl. Sci..

[38] Kutzman R., Wall H., Vinegar A. (1990). Toxic Hazards Research Unit, 1989.

[39] Weber L.W., Boll M., Stampfl A. (2003). Hepatotoxicity and mechanism of action of haloalkanes: Carbon tetrachloride as a toxicological model. Crit. Rev. Toxicol..

[40] Chowdhury G., Calcutt M.W., Nagy L.D., Guengerich F.P. (2012). Oxidation of methyl and ethyl nitrosamines by cytochrome P450 2E1 and 2B1. Biochemistry.

[41] Yaribeygi H., Panahi Y., Sahraei H., Johnston T.P., Sahebkar A. (2017). The impact of stress on body function: A review. EXCLI J.

[42] Sipes I.G., el Sisi A.E., Sim W.W., Mobley S.A., Earnest D.L. (1991). Reactive oxygen species in the progression of CCl4-induced liver injury. Adv. Exp. Med. Biol..

[43] Joung J.-Y., Cho J.-H., Kim Y.-H., Choi S.-H., Son C.-G. (2019). A literature review for the mechanisms of stress-induced liver injury. Brain Behav..

[44] Su L.J., Zhang J.H., Gomez H., Murugan R., Hong X., Xu D., Jiang F., Peng Z.Y. (2019). Reactive Oxygen Species-Induced Lipid Peroxidation in Apoptosis, Autophagy, and Ferroptosis. Oxid. Med. Cell. Longev..

[45] Poli G., Albano E., Dianzani M.U. (1987). The role of lipid peroxidation in liver damage. Chem. Phys. Lipids.

[46] El-Beshbishy H.A. (2005). The effect of dimethyl dimethoxy biphenyl dicarboxylate (DDB) against tamoxifen-induced liver injury in rats: DDB use is curative or protective. J. Biochem. Mol. Biol..

[47] Song H.Y., Ha K., Koh H., Shin I., Suh T. (1994). Effects of biphenyldimethyl dicarboxylate (DDB) on the lipid peroxidation, oxygen free radical scavenging enzymes activities and hepatic functions in ethanol-induced hepatotoxic rats. Korean J. Pharmacol..

[48] Duygu F., Karsen H., Aksoy N., Taskin A. (2012). Relationship of Oxidative Stress in Hepatitis B Infection Activity with HBV DNA and Fibrosis. Ann. Lab. Med..

[49] Chen Y., Dong H., Thompson D.C., Shertzer H.G., Nebert D.W., Vasiliou V. (2013). Glutathione defense mechanism in liver injury: Insights from animal models. Food Chem. Toxicol..

[50] (2017). ACMT Position Statement: Duration of Intravenous Acetylcysteine Therapy Following Acetaminophen Overdose. J. Med. Toxicol..

[51] Han D., Hanawa N., Saberi B., Kaplowitz N. (2006). Mechanisms of liver injury. III. Role of glutathione redox status in liver injury. Am. J. Physiol. Gastrointest. Liver Physiol..

[52] Weydert C.J., Cullen J.J. (2010). Measurement of superoxide dismutase, catalase and glutathione peroxidase in cultured cells and tissue. Nat. Protoc..

[53] Baker K., Marcus C.B., Huffman K., Kruk H., Malfroy B., Doctrow S.R. (1998). Synthetic combined superoxide dismutase/catalase mimetics are protective as a delayed treatment in a rat stroke model: A key role for reactive oxygen species in ischemic brain injury. J. Pharmacol. Exp. Ther..

[54] Rong Y., Doctrow S.R., Tocco G., Baudry M. (1999). EUK-134, a synthetic superoxide dismutase and catalase mimetic, prevents oxidative stress and attenuates kainate-induced neuropathology. Proc. Natl. Acad. Sci. USA.

[55] Nair A.B., Jacob S. (2016). A simple practice guide for dose conversion between animals and human. J. Basic Clin. Pharm..

[56] Birben E., Sahiner U.M., Sackesen C., Erzurum S., Kalayci O. (2012). Oxidative stress and antioxidant defense. World Allergy Organ. J..

[57] Wang C., Xu Y.Q. (2008). Diphenyl Dimethyl Bicarboxylate in the Treatment of Viral Hepatitis, Adjuvant or Curative?. Gastroenterol. Res..

